# Supplementary material for: In Vitro Neurotrophic Properties and Structural Characterization of a New Polysaccharide LTC-1 from Pyrola corbieri Levl (Luticao)
Source: Molecules. 2023 Feb 6;28(4):1544. doi: 10.3390/molecules28041544 (PMC9964326; doi:10.3390/molecules28041544)
Supplement: Supplementary file 1 [file molecules-28-01544-s001.zip › molecules-2188049-supplementary.pdf]

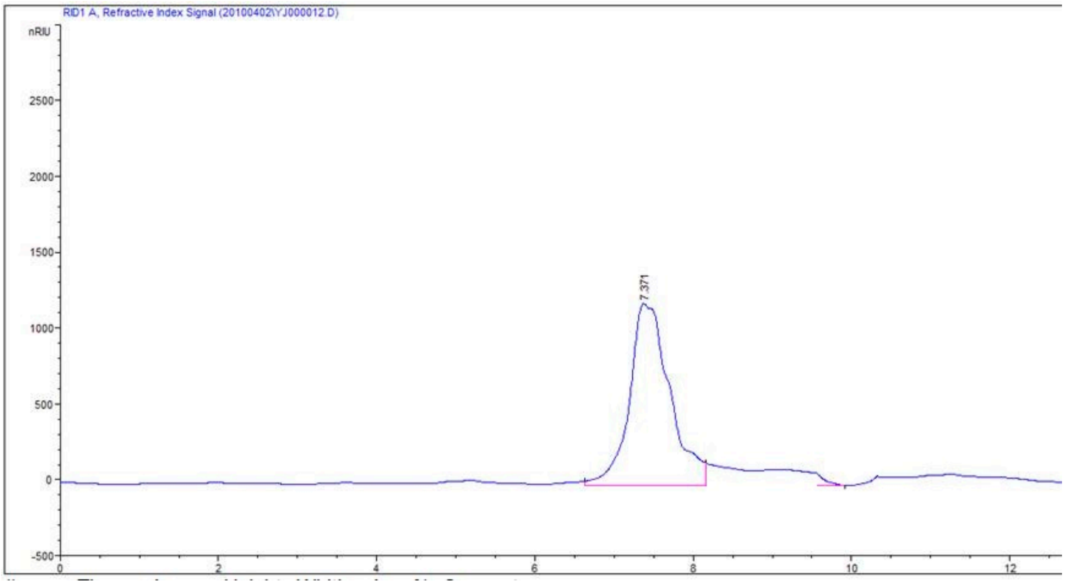

Figure S1. GPC analysis of LTC-1

Table S1 ： The result of LTC-1 periodate oxidation

sample	Reaction time（d）	Sample weighing（mmol）	Periodate consumption（mmol）	Periodate amount / hexose residue（mol/mol）	Formic acid consumption（mmol）	Formic acid / hexose residues（mol/mol）
LTC-1	14	0.12	0.13	1.10	0.03	0.27

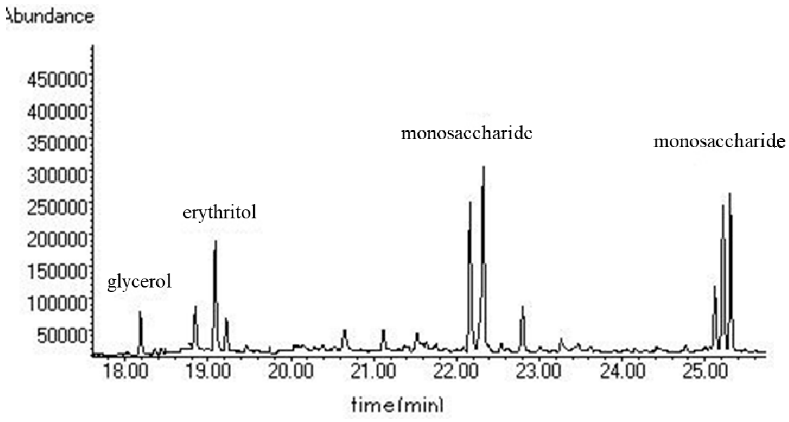

Figure S2. Part of GC-MS TIC from derivative of LTC-1 Smith degradation

Table S2: The result of LTC-1 partial hydrolysis

Monosaccharide composition	Rhamnose	Arabinose	Xylose	Mannose	Glucose	Galactose
II	-	-	-	+	+	+
III	-	+	+	+	+	+
IV	-	-	-	+	+	+

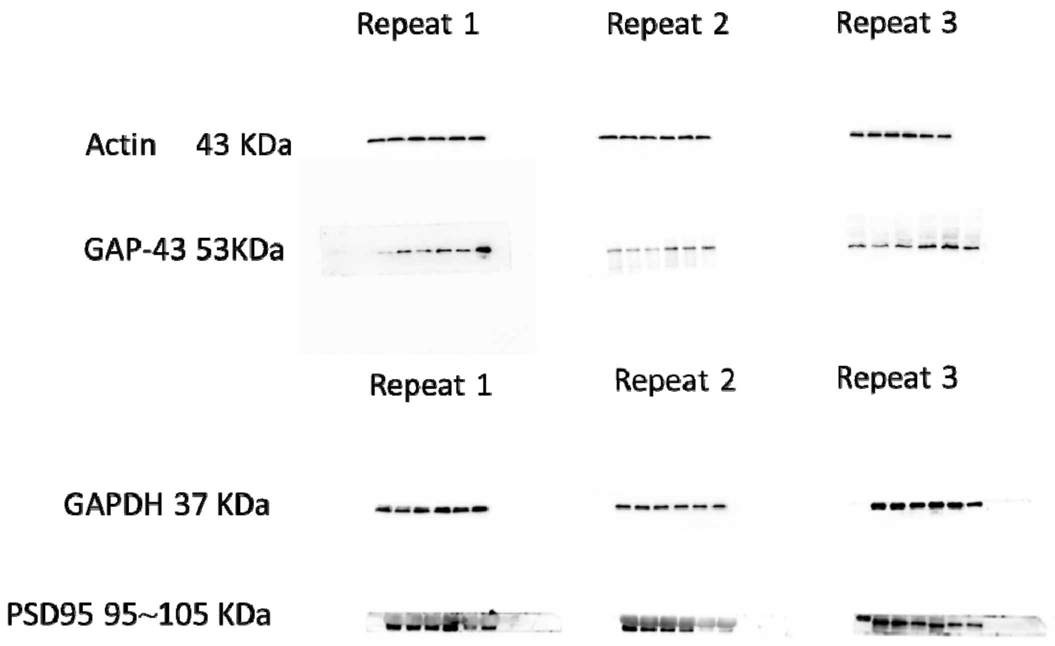

Figure S3. 0riginal western blot for three repeats
